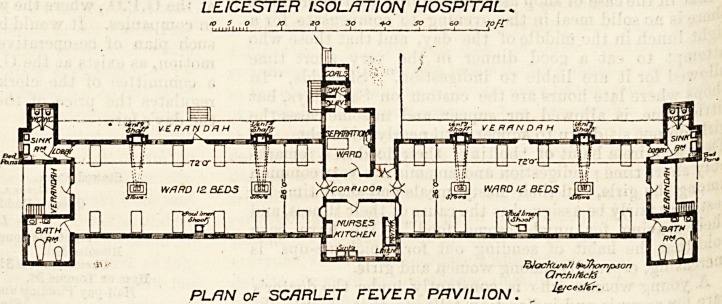# The New Isolation Hospital for Leicester

**Published:** 1901-09-14

**Authors:** 


### The Institutional Workshop.

### THE NEW ISOLATION HOSPITAL FOR LEICESTER.

This hospital was formally opened in 1900. It is situate near the town of Leicester on the Groby Road. The building consists of an administrative block; a laundry block, a discharging block, and of seven detached pavilions.

LEICESTER ISOLATION HOSPITAL.

BLOCK PLAN.

They are not connected by corridors, nor even by covered ways; but merely by roads so that the ambulance wagons can drive up to the entrance of any particular ward and leave the patient there.

Four of the pavilions are for scarlet fever, and all these blocks are only one storey high. Each pavilion consists of two wards between which are placed the ward kitchen, pantry, a connecting corridor, and a "separation ward" for one bed. A small sanitary block projects from this part of the pavilion, and close to it is an exit door.

Each ward is 72 feet long and 26 feet wide, and is intended for twelve beds. Each bed will therefore have a little more than 150 superficial feet allotted to it, and assuming a ceiling height of 14 feet the cubic space will be a little over 2,200 feet. An allowance which is ample provided the system of ventilation be good.

LEICESTER ISOLATION HOSPITAL.

PLAN OF SCARLET FEVER PAVILION.

Each bed has a window on both sides, and extraction flues are built in the outside walls for carrying away the vitiated air from the wards; but we are not told how these flues are constructed, nor what, if any, mechanical means is employed to ensure their being always in action irrespectively of inside or outside temperature, or motion of air.

At the extreme end of the ward are the projections for the bath-room and for the closets and sinks. The closet block is properly cut off by a ventilating passage, but the bath-room block is not. The latter arrangement is presumably with the object of preventing chill to patients returning from their baths. The sanitary towers containing the closets might have been better arranged had the walls between them and the verandah been placed in a line with the walls of the ward, and the walls, of course, ran out a corresponding distance. By these means cross-ventilating passages could easily have been obtained; it would then have been unnecessary to place the sinks opposite the closet doors. Both the linen-room and the pantry seem of small size for wards containing twenty-five beds. With these exceptions the wards are well planned and arranged.

One of the other pavilions is intended for typhoid cases, and it contains twenty-eight beds. It is similar to those above described, except that being for both men and women, it was necessary to provide two "separation wards." Verandahs are placed at the ends of the pavilions.

One of the remaining pavilions is for 12 beds. It is also of one storey, and is planned on the lines recommended by the Local Government Board. The other isolation pavilion is two storeys high. This block contains eight two-bedded rooms, and the accommodation is for 28 beds. The total accommodation at present is for 160 beds, but future extension is designed, and when built would raise the grand total to 240 beds.

The floors are of teak laid in narrow blocks herringbone fashion. The warming is, very properly, by open fireplaces; and these are assisted by low-pressure steam coils. It is stated that a separate apparatus is fitted up in each block for supplying hot water to the baths and lavatories. We believe that recent experiments have demonstrated beyond question the fact that if the correct principle be adopted, one furnace will supply hot water to any reasonable number of pavilions at any reasonable distance, and so save fuel and labour.

Electric lighting will be used. The discharging block is placed at the back of the porter's lodge, and is arranged in duplicate. The discharged patients will enter the block at one end in hospital clothes, will undress, bathe, put on other clothes, and leave by the opposite door.

The administration block contains the various offices, rooms for the medical staff and other officers, and for 20 nurses and 10 domestics.

The total cost will slightly exceed £54,000, being something like £340 a bed.

The architects were Messrs. Blackwell and Thomson.

## Figures and Tables

**Figure f1:**
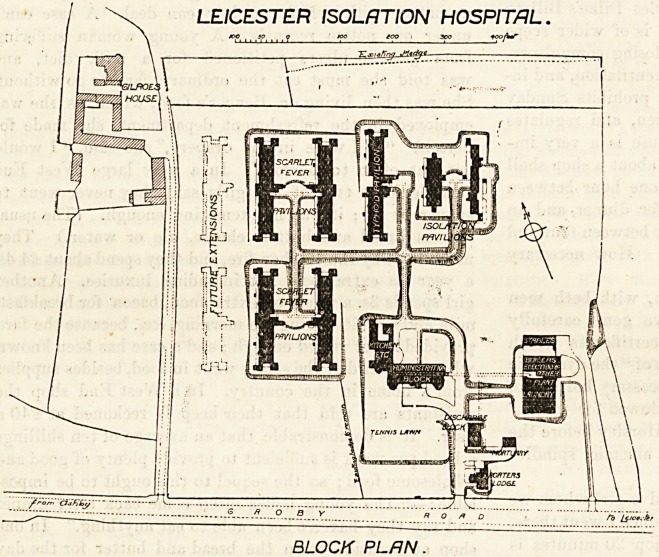


**Figure f2:**